# Liderazgo, determinantes sociales de la salud y equidad en la salud: el caso de Costa Rica^*^

**DOI:** 10.26633/RPSP.2021.101

**Published:** 2021-08-31

**Authors:** Epsy Campbell Barr, Michael Marmot

**Affiliations:** 1 Gobierno de la República de Costa Rica San José Costa Rica Gobierno de la República de Costa Rica, San José, Costa Rica.; 2 Institute of Health Equity, University College London Londres Reino Unido Institute of Health Equity, University College London, Londres, Reino Unido.

**Keywords:** Equidad en la salud, determinantes sociales de la salud, desarrollo humano, esperanza de vida, Costa Rica, Health equity, social determinants of health, human development, life expectancy, Costa Rica

## Abstract

Costa Rica es un país de especial interés en la Región de las Américas y en la salud mundial debido a su buena salud. El Programa de las Naciones Unidas para el Desarrollo clasifica a los países según su nivel de desarrollo humano con base en la esperanza de vida, la educación y el ingreso nacional. Aunque Costa Rica está clasificada en el puesto 63 y dentro del grupo “alto”, en términos de salud pertenece al grupo “muy alto”. En el 2018, la esperanza de vida media de los países del grupo “muy alto” era de 79,5 años, mientras que en Costa Rica era de 80. En el 2018, la mortalidad en menores de 5 años era de 8,8/1000 nacidos vivos, inferior a la de los países clasificados en el grupo de desarrollo humano “muy alto”. Los años de escolaridad previstos en Costa Rica ascienden a 15,4; más cercanos al promedio de 16,4 años del grupo de desarrollo humano “muy alto” que el promedio del grupo “alto”. El país es mucho más saludable de lo que podría predecirse por su ingreso nacional; más bien, es probable que otras características del desarrollo de la sociedad hayan desempeñado un papel fundamental en el desarrollo de la buena salud. Entre ellas figuran: *a*) la decisión de dejar de invertir en la defensa nacional, que liberó dinero para invertir en la salud, la educación y el bienestar de la población; *b*) la decisión de crear un sistema de salud universal financiado por el Estado, los empleadores y los trabajadores en el decenio de 1940; y *c*) el sistema educativo, que generó oportunidades para sacar de la pobreza a importantes sectores de la población, permitiéndoles disponer de condiciones sanitarias básicas que aumentan sus posibilidades de vivir más y mejor. A pesar de estos avances, persisten desigualdades en términos de ingresos y condiciones sociales, lo que plantea desafíos en el ámbito de la salud, en particular para los grupos de menores ingresos y los afrodescendientes e indígenas. Estas desigualdades deben abordarse mediante decisiones basadas en pruebas científicas, un mayor uso de datos desglosados que revelen los progresos realizados para hacer frente a esas desigualdades, y una mayor articulación del sector de la salud con las políticas que actúan sobre los determinantes sociales de la salud.

## Abstract (en)

Costa Rica has long been a country of special interest in the Americas and in global health because of its good health. The United Nations Development Programme ranks countries according to their level of human development based on life expectancy, education and national income. Although Costa Rica is ranked at 63 and classified as ‘High’, in terms of health it belongs in the ‘Very High’ group. In 2018 mean life expectancy for the ‘Very High’ countries was 79.5, while in Costa Rica it was 80. In 2018, under five mortality was 8.8/1000 live births, lower than countries ranked in the ‘Very High’ human development group. Expected years of schooling in Costa Rica is 15.4, closer to the average, 16.4 years, of the ‘Very High’ human development group than the average of the ‘High’ group. The country is much healthier than would be predicted by its national income; rather, other features of society’s development are likely to have played a key role in the development of good health. These include (i) the decision to cease investment in national defence, which freed up money to invest in health, education and the welfare of the population; (ii) the decision to create a universal health system financed by the State, employers and workers in the 1940s; and (iii) the educational system, that generated opportunities to lift important sectors of the population out of poverty, allowing them to have basic sanitary conditions that increase their possibilities to live longer and in better conditions. Despite these advances, inequalities in terms of income and social conditions persist, presenting challenges in the field of health, particularly for lower-income populations and those of African and indigenous descent. These inequalities must be addressed using decisions based on scientific evidence, a greater use of disaggregated data to reveal progress in addressing these inequalities, and with a broader articulation of the health sector with policies that act on the social determinants of health.

Costa Rica es un país de especial interés en la Región de las Américas y en la salud mundial debido a su buena salud. El Programa de las Naciones Unidas para el Desarrollo clasifica a los países según su nivel de desarrollo humano con base en la esperanza de vida, la educación y el ingreso nacional. En el informe del 2018, Noruega encabezaba la lista con el número 1 y Níger iba a la zaga con el 189 (*1*). Los primeros 58 países están clasificados como de “desarrollo humano muy alto”. Costa Rica, en el puesto 63, está clasificada como ”de desarrollo humano alto”, pero en términos de salud pertenece al grupo de “desarrollo humano muy alto”. En el 2018, la esperanza media de vida de los países del grupo “muy alto” era de 79,5 años. En Costa Rica era de 80 años, cuatro más que el promedio de 76 años para el grupo “alto”.

La buena salud de Costa Rica ilustra un punto importante sobre el desarrollo humano, en pocas palabras, que el dinero no lo es todo. Los países del grupo de desarrollo humano “bajo” (países con una esperanza media de vida baja, de 60,8 años) tuvieron en el 2018 un ingreso nacional bruto promedio per cápita de $2 521 ajustado según la paridad de poder adquisitivo (PPP), en comparación con $14 999 en el grupo de desarrollo humano “alto”, y $40 041 en el grupo “muy alto”. Si Costa Rica se clasificara solo por el ingreso nacional bruto per cápita, se ubicaría 15 lugares más abajo que el 63 que ocupa en el índice de desarrollo humano. Si se toma la esperanza de vida como criterio, Costa Rica se clasificaría 31 puestos más arriba. Es mucho más saludable de lo que cabría pronosticar por su ingreso nacional.

La buena salud de Costa Rica con niveles de ingreso nacional muy por debajo del promedio de los países del grupo de desarrollo humano “muy alto” ilustra un principio general. La Comisión de la Organización Panamericana de la Salud sobre Equidad y Desigualdades en Salud en las Américas llamó la atención sobre la curva de Preston que muestra una fuerte relación entre los ingresos nacionales y la esperanza de vida de los países con niveles bajos de ingreso nacional. Sin embargo, según el nivel de ingreso nacional de Costa Rica de $14 636 con PPP, es poca la relación entre el ingreso nacional y la esperanza de vida (*2*). Otras características del desarrollo de la sociedad son más importantes. De all el interés en analizar cómo Costa Rica logró su notable trayectoria de salud.

## EVOLUCIÓN DESDE LA CONSTITUCIÓN DE COSTA RICA DE 1949

Es probable que tres cambios notables hayan jugado un papel clave en el desarrollo de la buena salud en Costa Rica. En primer lugar, la decisión de dejar de invertir en la defensa nacional. Esto, al tiempo que envió una señal sobre las prioridades nacionales, liberó recursos para invertir en la salud, la educación y el bienestar de la población. Esta decisión fue como decir que Costa Rica es un país que invierte en el bienestar de su pueblo, no en el uso de los militares en su contra o en contra de otros.

La segunda política importante estuvo relacionada con una poderosa declaración de intenciones: la decisión de Costa Rica de crear un sistema de salud universal financiado por el Estado, los empleadores y los trabajadores en la década de 1940. El sistema de salud costarricense se basa en la solidaridad y requiere que el Estado costarricense invierta en la salud, pero también exige una responsabilidad conjunta a todos los actores de las esferas pública y privada, trabajadores y empleadores. La creación de la Caja Costarricense de Seguro Social hace que el país se destaque de manera especial en la provisión de cobertura pública de salud.

En tercer lugar, el sistema educativo generó importantes oportunidades para sacar a importantes sectores de la población de la pobreza, lo que les permitió tener condiciones sanitarias básicas que aumentan sus posibilidades no sólo de vivir más tiempo en años, sino también de vivir en mejores condiciones. La educación brindó oportunidades para la promoción de la salud.

Costa Rica se ve a sí misma como un país en desarrollo. Ya nos hemos referido a las cifras de esperanza de vida que, de por sí, ya calificarían para que el país estuviera en el grupo de desarrollo humano “muy alto”. Las tasas de mortalidad de menores de 5 años en todo el mundo van desde 2/1000 nacidos vivos en Islandia hasta 127 en Chad. En el 2018, la mortalidad de menores de 5 años en Costa Rica fue de 8,8/1000 nacidos vivos, inferior a la de países como Uruguay y Argentina, que se encuentran en el grupo de desarrollo humano “muy alto”. Los años de instrucción previstos en Costa Rica ascienden a 15,4; más cerca del promedio de 16,4 años del grupo de desarrollo humano “muy alto” que del promedio del grupo de desarrollo humano “alto”. En los aspectos importantes para el bienestar humano, la educación y la salud, Costa Rica es un éxito.

## EL DESAFÍO DE LAS DESIGUALDADES

Al igual que con la mayoría de los países de América Latina, Costa Rica está marcada por grandes desigualdades en lo que respecta a los ingresos y las condiciones sociales. Esto plantea desafíos para la salud, lo que incluye peor salud para los pobres y para las personas afrodescendientes. Estas desventajas sanitarias, así como las de la población indígena, se observan en toda la Región.

Todos los países se enfrentan a estos problemas de inequidad en la salud. El Gobierno de Costa Rica promovió una serie de medidas que han tenido un impacto general en la población. La primera gran decisión fue poner el tema de la salud de los residentes en el centro del debate. Es decir, que todas las decisiones económicas estaban sujetas al escrutinio de su repercusión en las cuestiones de salud. El Gobierno de Costa Rica pudo hacerlo precisamente por su tradición de contar con un sistema de salud robusto. En segundo lugar, y esto no debería necesitar comentarios, las decisiones se basan en la ciencia, con información empírica fiable, algo que ha sido un elemento absolutamente central. En adelante, se centrará más la atención en los resultados desglosados para observar los avances en la lucha contra las desigualdades en la salud. Es muy lamentable que basar las políticas en fundamentos científicos sólidos sea algo que los países no practiquen universalmente. En tercer lugar, el Gobierno ha tomado medidas de acción afirmativa a favor de las mujeres. Se ha logrado disminuir las tasas de embarazo adolescente, aunque el exceso de adolescentes afrodescendientes en esta situación sigue siendo un desafío particular. Es probable que esto esté relacionado con las tasas de pobreza altas en las niñas y mujeres afrodescendientes. En cuarto lugar, la salud mental está definitivamente en la agenda. Se han tomado una serie de decisiones que se centran fundamentalmente en recomendaciones a las personas y las familias para que el tema de la salud mental no quede fuera de la conversación sobre la salud integral.

En relación con lo anterior, Costa Rica ha promovido dos protocolos: uno dirigido específicamente a la población indígena y otro a la población afrodescendiente. Se trata de directrices para el sector de la salud hacia la población indígena y directrices para la población afrodescendiente. Los progresos también requerirán que al sector de la salud se sumen políticas inclusivas relativas a determinantes sociales de la salud en general.

## COLABORACIÓN PARA HACER FRENTE A LA PANDEMIA DE COVID-19

La Caja Costarricense de Seguro Social y el Ministerio de Salud han sido dos instituciones clave para gestionar la pandemia. Como declaración tanto de principios como de práctica, ambas instituciones afirmaron que las personas no son atendidas de acuerdo con la nacionalidad, sino por las necesidades de salud. Este es un elemento de gran relevancia porque Costa Rica es un país donde hay una considerable población extranjera vinculada a la producción agrícola, la construcción y los servicios domésticos, pero que no necesariamente tienen seguro médico. Es por eso que la primera decisión fue no generar discriminación alguna en el acceso a los servicios de salud.

Otras instituciones que han colaborado son el Instituto Mixto de Ayuda Social (IMAS) y la Comisión Nacional de Emergencias (CNE), que coordina todas las medidas ante la emergencia sanitaria. Por debajo de la CNE están todas las instituciones estratégicas como el Instituto Nacional de la Mujer, el Patronato Nacional de la Infancia, el Instituto Nacional de Seguros, un gran número de empresas públicas como el Instituto Costarricense de Acueductos y Alcantarillados y el Instituto Costarricense de Electricidad. Una cuestión muy importante es el acceso al agua potable y hay un compromiso de las empresas públicas de no suspender los servicios durante esta emergencia nacional.

El Instituto Costarricense de Electricidad ha comenzado a prestar servicios como plataforma de información para que las personas puedan tener información precisa sobre temas relacionados con la pandemia y la Fábrica Nacional de Licores, que también es una empresa pública, se ha convertido en el mayor proveedor de alcohol y soluciones antisépticas para toda la población, a precios asequibles, como elemento central para la higiene.

El Ministerio de Seguridad Pública ha estado protegiendo permanentemente las fronteras, con el fin de instalar una valla sanitaria para cuidar la salud pública de todas las personas que viven en Costa Rica y no permitir la entrada irregular.

Esta acción interinstitucional requiere coordinación y la Comisión Nacional de Emergencias ha proporcionado el liderazgo a través de un comité de alto nivel toma decisiones a diario, y está facultada para invertir una gran cantidad de recursos económicos y transferir ayuda humanitaria.

A través de esta Comisión operativa se supervisa la respuesta de salud que debe darse a nivel territorial y nacional. La Comisión, junto con el Ministerio de Salud, han definido claramente el liderazgo en esta cuestión. Esta estructura permite respuestas efectivas a una realidad que, claramente, fue inesperada.

## EL SISTEMA DE SALUD Y EL PÚBLICO

Los ciudadanos costarricenses reconocen a las instituciones de salud como las más relevantes y fuertes del país, a pesar de que es posible que no hayan acudido a los servicios de salud como resultado de la pandemia. Este reconocimiento es merecido. No nació como resultado de la pandemia, sino que es el resultado de la larga y dedicada labor realizada por estas instituciones en el país.

La pandemia ha acentuado la necesidad de información. El Gobierno ha realizado conferencias de prensa todos los días desde el 6 de marzo cuando se detectó el primer caso de COVID-19 en el país. El público puede acceder a la conferencia de prensa a través de las páginas de redes sociales del Gobierno de Costa Rica, junto con la información publicada que las personas pueden consultar en cualquier momento.

Una parte de esta información también se transmite a través de los medios de comunicación, lo que contribuye a educar a la población. Esta comunicación, en lugar de sembrar el miedo, hace que la población costarricense esté más informada, lo que les permite tomar decisiones que son importantes para su propia salud y para la de la comunidad.

## DESARROLLO SOSTENIBLE

Las medidas para mejorar la salud y reducir las desigualdades están en consonancia con los Objetivos de Desarrollo Sostenible. De importancia central, Costa Rica ha presentado un ambicioso Plan Nacional de Descarbonización para convertir el 100% del uso de combustibles fósiles en energía renovable. Hoy en día, en términos de electricidad, el país ya es prácticamente autónomo y depende principalmente de energías limpias. El siguiente reto para la descarbonización es el transporte, uno de los elementos en los que Costa Rica está trabajando actualmente.

El Plan Nacional de Salud requiere una respuesta absolutamente clara a situaciones como la pandemia y los problemas asociados. Es necesario invertir en educación para la salud. Ciertamente, la educación del público ha ayudado a las autoridades de salud a tener un enfoque científicamente responsable de esta pandemia.

## ENSEÑANZAS DE COSTA RICA PARA OTROS PAÍSES DE LA REGIÓN

Costa Rica es sumamente humilde y preferiría reflexionar y reafirmar lo que ha hecho en lugar de recetar a otros con diferentes historias y realidades lo que deben hacer. La experiencia de Costa Rica muestra que después de alcanzar un umbral de crecimiento económico, no es necesario un mayor crecimiento económico para lograr el éxito en la salud. La esperanza de vida de Costa Rica, de 80 años (cifras del PNUD del 2018), la sitúa en un punto medio del rango de países calificados como de desarrollo “muy alto”.

La decisión en la década de 1940 de abolir las fuerzas armadas e invertir en agua potable, educación y atención de la salud, y bienestar de la población ha sido de gran importancia para la mejora de la salud de la población.

En ese contexto, el desarrollo del sistema de atención de la salud ha sido vital. Las políticas de salud han estado en el centro de las políticas de desarrollo. Costa Rica tiene un sistema de salud solidario, donde el trabajo se hace no solo en la atención inmediata, sino también por niveles. El primer nivel parte de la lógica de contener y abordar los problemas de salud antes de que los pacientes requieran hospitalización. Cientos de equipos de atención básica en todo el país están en las comunidades y buscan generar registros de salud de las personas. Este trabajo facilita enormemente el enfoque en tiempos de pandemia.

## Declaración

Las opiniones expresadas en este manuscrito son responsabilidad del autor y no reflejan necesariamente los criterios ni la política de la *RPSP/PAJPH* y/o de la OPS.
